# Wafer-Scale Fabrication of Sub-10 nm TiO_2_-Ga_2_O_3_*n-p* Heterojunctions with Efficient Photocatalytic Activity by Atomic Layer Deposition

**DOI:** 10.1186/s11671-019-2991-1

**Published:** 2019-05-14

**Authors:** Hongyan Xu, Feng Han, Chengkai Xia, Siyan Wang, Ranish M. Ramachandran, Christophe Detavernier, Minsong Wei, Liwei Lin, Serge Zhuiykov

**Affiliations:** 1 School of Materials Science and Engineering, North University of China, Taiyuan, 030051 People’s Republic of China; 2 Department of Solid State Science, Ghent University, Krijgslaan 281/S1, B-9000 Ghent, Belgium; 3 Berkeley Sensor and Actuator Center, Department of Mechanical Engineering, University of California, Berkeley, CA 94720 USA; 4 Ghent University Global Campus, 119 Songdomunhwa-ro, Yeonsu-gu, Incheon, 21985 South Korea

**Keywords:** TiO_2_-Ga_2_O_3_, *n-p* heterostructures, atomic layer deposition, 2D semiconductors

## Abstract

**Abstract:**

Wafer-scale, conformal, two-dimensional (2D) TiO_2_-Ga_2_O_3_ *n-p* heterostructures with a thickness of less than 10 nm were fabricated on the Si/SiO_2_ substrates by the atomic layer deposition (ALD) technique for the first time with subsequent post-deposition annealing at a temperature of 250 °C. The best deposition parameters were established. The structure and morphology of 2D TiO_2_-Ga_2_O_3_ *n-p* heterostructures were characterized by the scanning electron microscopy (SEM), X-ray photoelectron spectroscopy (XPS), electrochemical impedance spectroscopy (EIS), etc. 2D TiO_2_-Ga_2_O_3_ *n-p* heterostructures demonstrated efficient photocatalytic activity towards methyl orange (MO) degradation at the UV light (*λ* = 254 nm) irradiation. The improvement of TiO_2_-Ga_2_O_3_ *n-p* heterostructure capabilities is due to the development of the defects on Ga_2_O_3_-TiO_2_ interface, which were able to trap electrons faster.

**Graphical Abstract:**

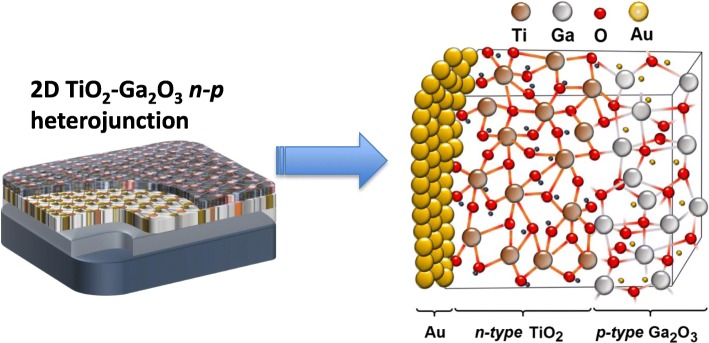

## Background

Fabrication of 2D *p-n* heterojunctions of semiconductor oxides is one of the key directions of future development of nanostructures with unique distinguishable properties, as they are able to combine various outstanding features of both semiconductors at the nanoscale [1–4]. However, it is extremely challenging to fabricate them defects-free over the wafer area, particularly when the thickness of each oxide is only few nanometers [2]. In order to overcome numerous manufacturing challenges, ALD technology has already established clear and unprecedented advantages in the development of conformal nano- and monolayers of the semiconductor oxides and their 2D heterostructures with the thickness less than 10 nm on wafer-scale with high aspect ratio [3, 5–7]. In addition, various new approaches were also initiated recently for the development of 2D heterostructures with enhanced functional capabilities [8–11]. They specifically targeted both oxygen evolution reaction (OER) and hydrogen evolution reaction (HER), as a core processes for various renewable energy systems [12]. However, in comparison to HER, OER with multistep, four-electron process evolved is severely constrained by its sluggish kinetics [13]. Thus, more efforts have therefore been devoted to improve the conductivity of heterostructures and control the electronic structures of their surface active sites through the modulation of their morphology, constituent compositions, and/or dopants [8, 12]. Moreover, regulating the surface-adsorbed species may also provide an alternative valuable approach to fine-tuning the interfacial properties, particular at nanostructured heterojunctions, and the electronic structures of active materials [14].

More importantly, it was demonstrated that the decreasing free energy of the OER intermediates at the nano-interface would remarkably enhance the inherent electrochemical performance of catalyst [13]. In this regard, surface engineering is well illustrated to improve the accessibility of the reactants and to alter the electrochemical activity of the catalysts [14]. To achieve such enhancements of electrochemical properties of nanostructured heterostructures, various technological approaches have been utilized. Among them, the ALD technique can be used to deposit wafer-scaled nanomaterials with controlling their deposition rate at the Angstrom scale. Additional vital advantage of ALD is its self-limited nature by depositing materials in an atomic layer-to-layer [5, 6].

The alternative approach represents the development of 2D C-MOFs via the combination of “through-space” and “through-bind” strategies [14]. In particular, hexahydroxytriphenylene ligand-based 2D C-MOFs possess M-O4 (M–transitional metals) as their secondary building units and provide discrete metal-replicable layers as promising reactive sites for OER [14]. Moreover, these C-MOFs can remain stable in high pH solution, which is quite important for OER. Thus, all these above-mentioned recent advancements indirectly confirmed that no other technologies of making 2D nanostructures, including *sol-gel*, chemical vapor deposition (CVD), RF sputtering, etc., are capable to deliver uniformed deposition at the Ångstrom level over the large areas of Si/SiO_2_ wafers with precise control of the deposition rate and thickness. Therefore, most of the developed recipes for ALD of 2D nanostructures using specific precursors possess valuable *know-how* and represent a highly repeatable process on the semi-industrial scale [5, 15, 16].

One of the main 2D semiconductors successfully utilized in the different photovoltaic applications is titanium dioxide (TiO_2_), which is a typical *n*-type semiconductor with wide bandgap *E*_g_ = ~3.2 eV [5, 15–18]. There are numerous scientific reports focused on the different approaches for improvement of its properties such as changing thickness of nanostructured 2D TiO_2_ down to monolayer [15, 16], doping TiO_2_ by other nanostructures semiconductors [5, 17], surface functionalization of 2D TiO_2_ [18] and making *n-p* heterojunctions [19]. In addition, low electron/hole recombination is blamed for the low quantum yields, which is still a big obstacle for the improvement of photocatalytic activity. Therefore, fabrication of efficient *n-p* heterojunctions has been proposed and attempted with the different levels of success during the last few years [4, 17, 20–22]. Specifically, it was found that the fabricated *n-p* heterojunctions could sufficiently reduce the recombination rate of the photo-generated electron/hole pairs with the following enhancement of the overall photocatalytic activity [1, 23, 24]. Thus, the combination of *p-* and *n-*type semiconductor oxides has paved the way for the further development of *n-p* heterojunctions and optimization of their photocatalytic capabilities [25].

In this regard, 2D surface functionalization of 2D *n*-type TiO_2_ by ALD of another *p*-type semiconductor on the top of TiO_2_ represents a unique strategy of making *n-p* heterojunctions and combining various outstanding properties of both semiconductors [5]. On the other hand, semiconductor oxides with a d^10^ electron configuration have recently attracted considerable attention for their superior activities as potential dopant. This is mainly owing to their conduction bands being formed by hybridized *sp* orbits with a large dispersion, which enabled them to generate electrons with the large mobility [26]. Gallium oxide (Ga_2_O_3_), as a typical representative of such d^10^ semiconductor oxides, belongs to the group of transparent semiconducting oxides with a wide band gap and electrical conductivity. It exhibits the largest band gap with *E*_g_ = 4.8 eV and thus a unique transparency from the visible into the UV region and good luminescence properties [27]. *β*-Ga_2_O_3_ is reported to be the most stable polymorph among five existing polymorphs of Ga_2_O_3_ within the high-temperature range [28]. Moreover, nontoxic β-Ga_2_O_3_ displayed significant potential for photocatalytic air purification, particularly for the elimination of toxic aromatic compounds [29]. Therefore, all these distinguishable properties of β-Ga_2_O_3_ [30] substantiated a lot of efforts for the best suitable technologies of Ga_2_O_3_ deposition at the nanoscale [31–33].

Notwithstanding the great attempts dedicated to the ALD of 2D semiconductor oxides during last few years, authors wish to stress that so far 2D TiO_2_-Ga_2_O_3_ *n-p* heterostructures with the thickness less than 10 nm have not yet been reported. In this work, 2D TiO_2_-Ga_2_O_3_ *n-p* heterostructures were ALD-fabricated on wafer-scale for the first time using Ti(N(CH_3_)_2_)_4_ and C_33_H_57_GaO_6_ as TiO_2_ and Ga_2_O_3_ precursors, respectively. Their optimal deposition parameters were established and structural and photocatalytic properties were investigated.

## Results and Discussion

**Fig. 1:**
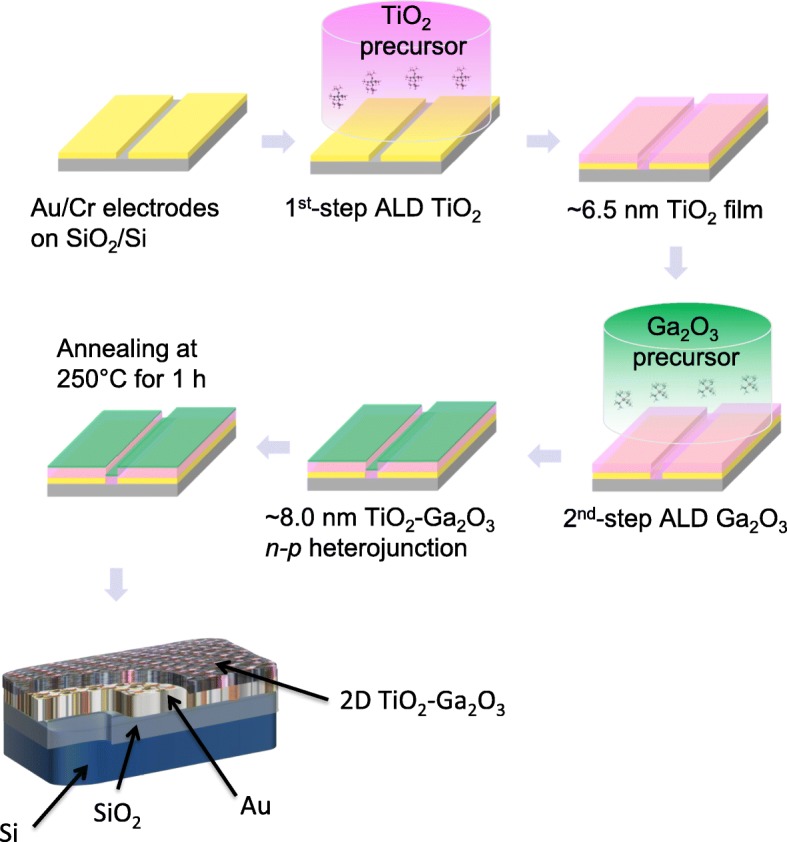
Schematic fabrication process of 2D TiO_2_-Ga_2_O_3_ *n-p* heterostructures

**Fig. 2:**
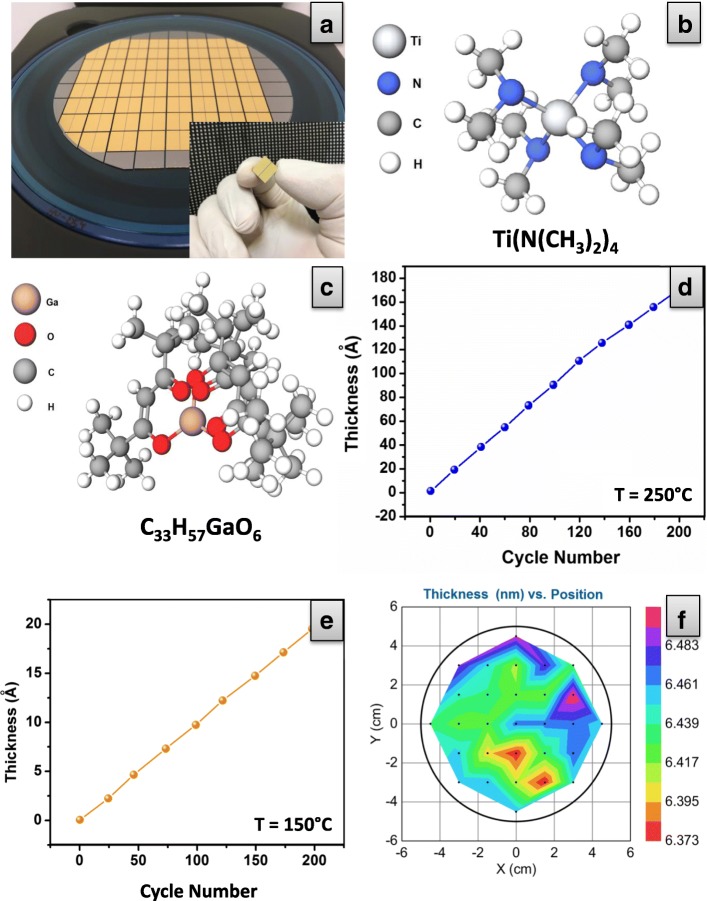
**a** The optical image of wafer-scale ALD-deposited TiO_2_-Ga_2_O_3_ *n-p* heterostructures films, insert—an individual 1 cm^2^ electrode. **b**, **c** Graphical scheme of chemical formula of Ti(N(CH_3_)_2_)_4_ and C_33_H_57_GaO_6_ precursors, respectively. **d**, **e** The graph of thickness versus ALD cycle number of TiO_2_ and graph of thickness versus ALD cycle of Ga_2_O_3_ films, respectively. **f** The spectroscopic ellipsometry mapping of thickness of 2D TiO_2_ film

Figure 1 illustrates the fabrication process of 2D TiO_2_-Ga_2_O_3_ *n-p* heterostructures on the Si/SiO_2_ substrate. Figure 2 schematically depicts the details of ALD depositions. After depositions, wafers were diced on 1.0 × 1.0 cm segments (Fig. 2a) for further testing. For ALD 2D TiO_2_-Ga_2_O_3_ *n-p* heterostructures Ti(N(CH_3_)_2_)_4_ and C_33_H_57_GaO_6_ (Strem Chemicals Inc., USA) were used as TiO_2_ and Ga_2_O_3_ precursors, respectively. Their graphical interpretations are given in Fig. 2b, c. The growth per cycle (GPC) yielded from the slopes of growth curves shown in Fig. 2d, e was calculated to be around 0.7 Å/cycle and 0.16 Å/cycle for TiO_2_ and Ga_2_O_3_, respectively. The growth curves were linear without any nucleation delay for both TiO_2_ and Ga_2_O_3_ samples, implying that the self-limited property of ALD growth process and the film thickness could be developed precisely by varying the number of ALD cycles. The lower growth rate of 2D Ga_2_O_3_ nano-films makes its applications on the doping and modification possible [34]. Noteworthy, the optimal ALD deposition parameters for each precursor are usually established after several initial trials [6]. After each deposition cycle the variable angle in situ ellipsometry measurements (J.A. Woollam M2000 DI) were carried to monitor the uniformity and to measure the thickness of films. For example, Fig. 2f illustrates the in situ ellipsometry measurements for 2D TiO_2_ with the average thickness of ~ 6.45 nm. Since the thickness measurements were found difficult on heterostructure, the Ga_2_O_3_ film growth was followed, using in situ ellipsometry measurement, on SiO_2_/Si substrate that was placed on the heater block, together with the sample. After the deposition, the Ga_2_O_3_ film thickness on heterostructure was confirmed by comparing the amount of material deposited on it and the reference SiO_2_/Si using X-ray fluorescence measurements [19]. 2D Ga_2_O_3_ films had an average thickness of ~ 1.5 nm, which resulted in the total thickness of 2D TiO_2_-Ga_2_O_3_ heterostructures to be ~ 8.0 nm. All fabricated samples were subsequently annealed in the air for 1 h at 250 °C with a heating rate of 0.5 °C/min.

**Fig. 3:**
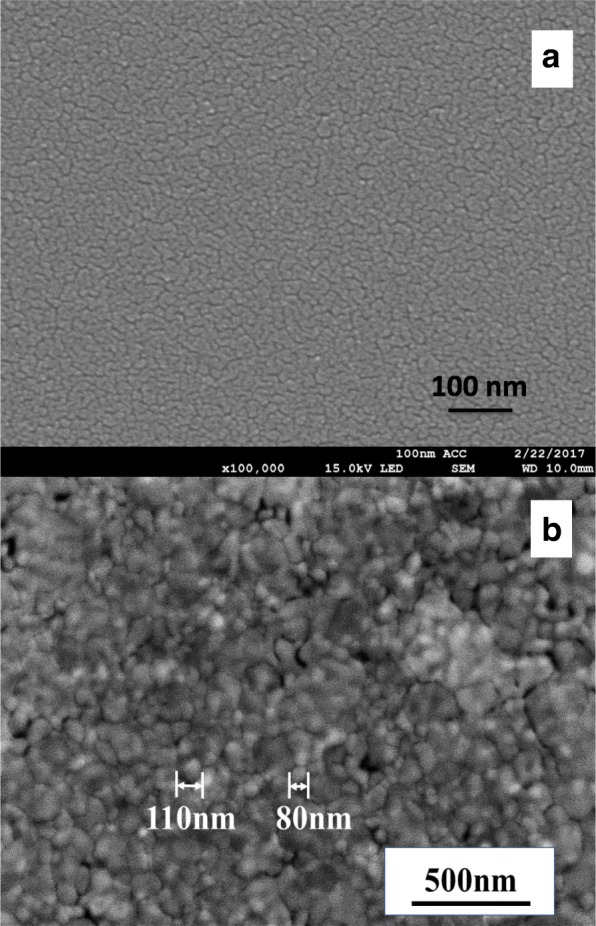
SEM images of the ALD-deposited 2D (**a**) TiO_2_ and (**b**) TiO_2_-Ga_2_O_3_ heterostructure nano-films

Figure 3 shows SEM surface morphology images for both ALD-fabricated 2D TiO_2_ (thickness ~ 6.5 nm) and Ga_2_O_3_ (thickness ~ 1.5 nm) nano-films. It is noteworthy that the TiO_2_ nano-grains in the fabricated films were uniformly distributed over Si/SiO_2_ wafer and varied in size from approximately ~ 30 to ~ 70 nm prior to Ga_2_O_3_ deposition. Figure 3a depicts surface morphology of TiO_2_ nano-film consisting of the flat nano-particles. Then, the ALD-developed ~ 1.5-nm-thick Ga_2_O_3_ nano-films were fabricated on the top of ~ 6.5-nm-thick TiO_2_ nano-films. The ALD-developed sub-10 nm Ga_2_O_3_-TiO_2_ heterostructures were subsequently annealed at 250 °C. Thus, Fig. 3b depicts crystalline surface morphology of the Ga_2_O_3_ in heterostructure after annealing. The Ga_2_O_3_ nano-film consists of uniformly distributed Ga_2_O_3_ nano-grains with the average size from ~ 80 to ~ 110 nm. Owing to the extremely thin nature of the ALD-fabricated nano-films, employment of the X-ray diffraction technique for crystallinity investigation of these films was not possible.

**Fig. 4:**
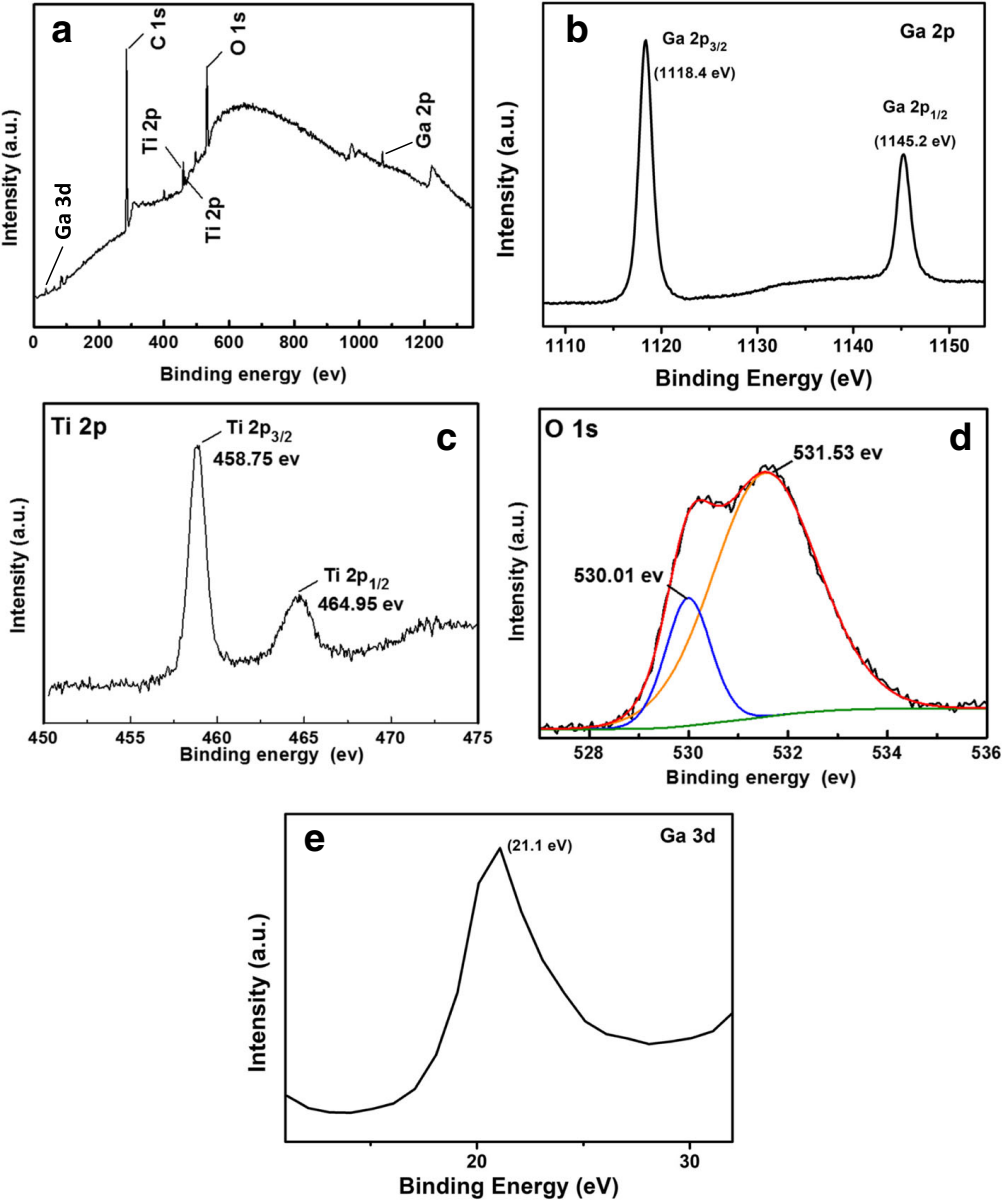
XPS spectra of 2D TiO_2_-Ga_2_O_3_ *n-p* heterostructures. **a** Full survey scan spectrum. **b** Ga 2p region. **c** Ti 2p region. **d** O 1s region. and **e** Ga 3d region

Chemical composition and bonding states of 2D TiO_2_-Ga_2_O_3_ heterostructures were studied by XPS with Fig. 4a representing the TiO_2_-Ga_2_O_3_ heterostructure scan survey. The charge shift spectrum was calibrated for C1s peak at 284.8 eV. Three main elements of Ti, O, and Ga are clearly observed. In addition, C1s peak was also detected as it was originated from the reference to calibrate the binding energies of the peaks. Figure 4b depicts high-resolution two quasi-symmetrical Ga 2p_1/2_ and Ga 2p_3/2_ peaks for Ga-O bonding at 1145.2 eV and 1118.4 eV with a separation distance of 26.8 eV, which is consistent with the binding energy of Ga 2p for doped *β*-Ga_2_O_3_ [35, 36]. The weak energy peak for Ga 3d is centered at 21.1 eV, which is caused by the presence of Ga-O bond reported for *p*-type *β*-Ga_2_O_3_ films [37], but not observed for the *n-*type *β*-Ga_2_O_3_ structures [38]. The Ga 3d peak is asymmetrical, which was ascribed to the hybridization of Ga 3d and O 2s states near the valence band [39]. Figure 4c displayed the high-resolution scan of Ti 2p. The doublet peaks demonstrated in Fig. 4c correspond to Ti 2p_3/2_ and Ti 2p_1/2_ with the spin-orbital splitting of 6.2 eV, which were attributed to Ti^+4^ oxidation state. It should be noted that the obtained XPS results in this investigation are slightly different from our previous report on the development of TiO_2_ monolayer [15] and bi-layer [3] grown by ALD. This difference is reasonable considering the amount of Ti in the samples.

The O 1s peak in the XPS spectrum (Fig. 4d) could be deconvoluted into two major peaks. The main binding energy component centered at 531.53 eV is attributed to oxygen vacancies or OH^-1^ adsorbed species on the surface [38]. The second binding energy peak at 530.01 eV can be the characteristic of the lattice oxygen in the TiO_2_-Ga_2_O_3_ heterostructure. Very relevant to this investigation was our previous study on ALD TiO_2_ bi-layer confirming the influence of SiO_2_ substrate, where the bottom oxygen of TiO_2_ is shared with SiO_2_ making 2D TiO_2_ slightly non-stoichiometric [3]. Thus, this non-stoichiometry plays a critical role in 2D TiO_2_-Ga_2_O_3_ heterostructure while the thickness of Ga_2_O_3_ ALD on the top of TiO_2_ is only ~ 1.5 nm. The enlarged energy peak for Ga 3d is presented in Fig 4e. Presence of Ga 3d peak in the spectrum is confirmation of the *p*-type conductivity for Ga_2_O_3_ in the heterostructure, as being reported [37]. For further investigation of the conductivity type of 2D β–Ga_2_O_3_, additional 4.8-nm-thick Ga_2_O_3_ samples were subjected to the Hall coefficient measurements at *T* = 25 °C. The measured Hall coefficient value of 8.292 × 10^4^ cm^3^/C independently confirmed the stable *p*-type performance of 2D Ga_2_O_3_.

**Fig. 5:**
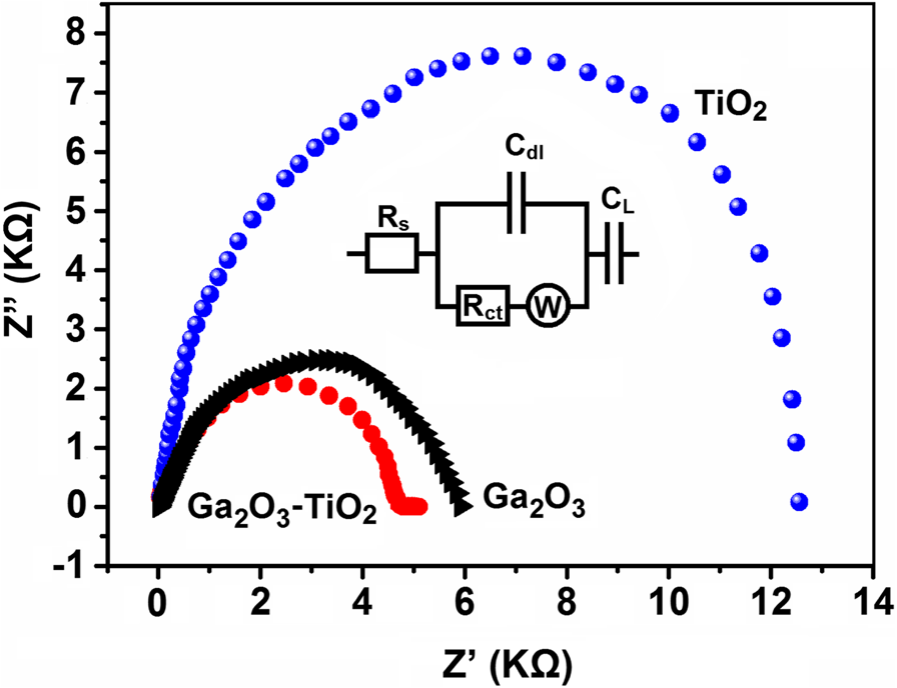
Nyquist plots of the 2D TiO_2_, Ga_2_O_3_, and TiO_2_-Ga_2_O_3_ heterostructures tested in air at a temperature of 25 °C

Figure 5 expresses the plotted EIM measurements of the spectra for 2D TiO_2_ (~ 3.5 nm), Ga_2_O_3_ (~ 3.5 nm), and 2D TiO_2_-Ga_2_O_3_ heterostructures (~ 8.0 nm), respectively. EIS measurements were carried out in air at the temperature of 25 °C and the impedance results were obtained using the Randles equivalent circuit. It is noteworthy that the fitted Nyquist plots in Fig. 5 revealed the charge-transfer resistance (*R*_ct_ = 4.5 kΩ) of 2D TiO_2_-Ga_2_O_3_ heterostructures with a thickness of ~ 8.0 nm being about 2.7-fold lower than that of ALD-developed 2D TiO_2_ (*R*_et_ = ~ 12.5 kΩ) and even slightly lower than that of 2D Ga_2_O_3_ (*R*_et_ = ~ 6.0 kΩ). This fact further designates that 2D TiO_2_-Ga_2_O_3_ heterostructures possess a much faster charge-transfer characteristics than that of 2D TiO_2_ and Ga_2_O_3_. Although the measured impedance value for of Ga_2_O_3_ was slightly higher than the reported value for 2D ALD-fabricated Ga_2_O_3_ [40], this was partially due to the sub-nanometer thickness of the Ga_2_O_3_ film [40] compare to the ~ 3.5-nm-thick Ga_2_O_3_ in our experiments and was also partially owing to the fact that the developed 2D Ga_2_O_3_ was not fully crystallized at the annealing temperature of 250 °C.

**Fig. 6:**
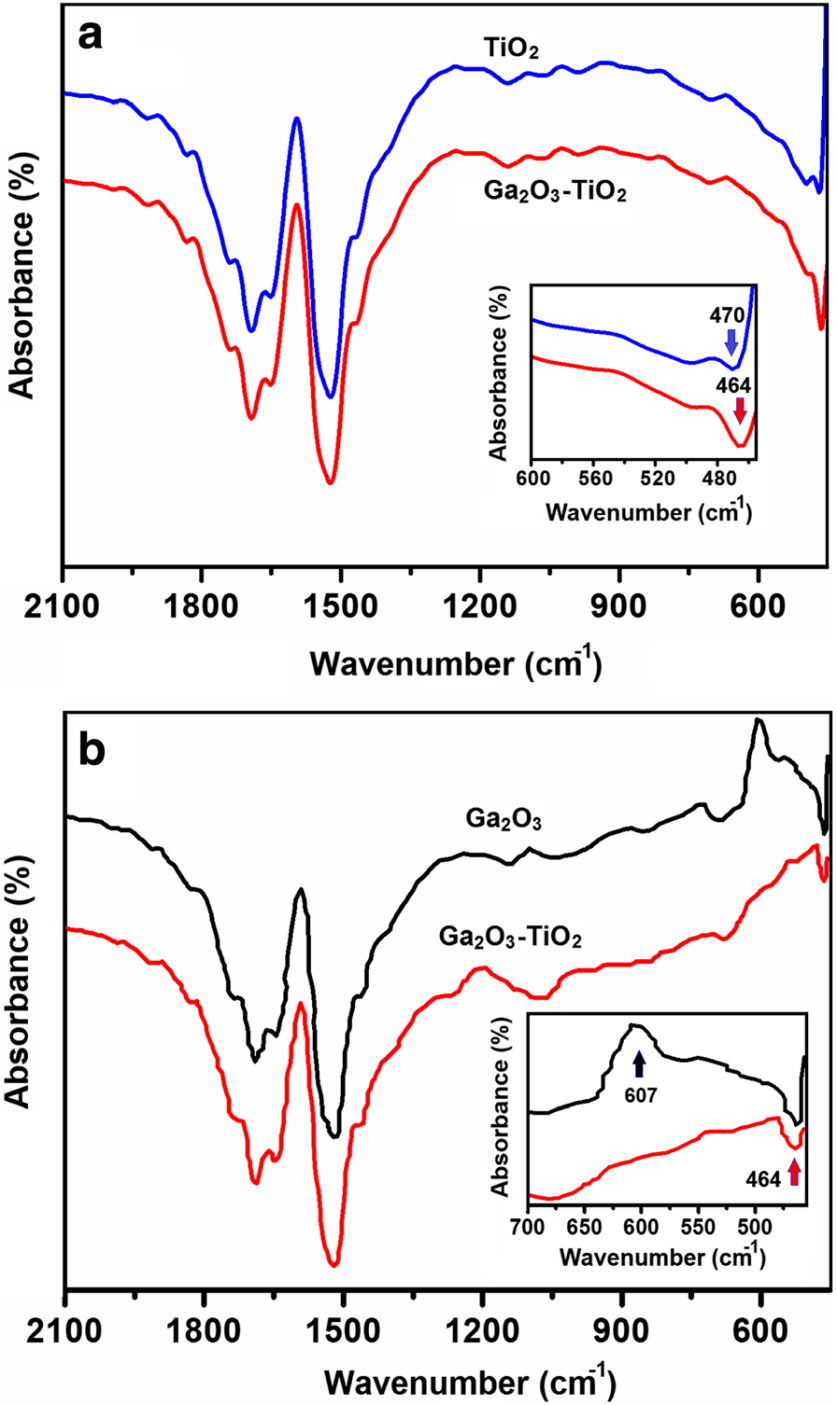
FTIR spectra of ALD-fabricated 2D TiO_2_ and TiO_2_-Ga_2_O_3_ *n-p* heterostructures (**a**) and 2D Ga_2_O_3_ and TiO_2_-Ga_2_O_3_ *n-p* heterostructures (**b**)

**Fig. 7:**
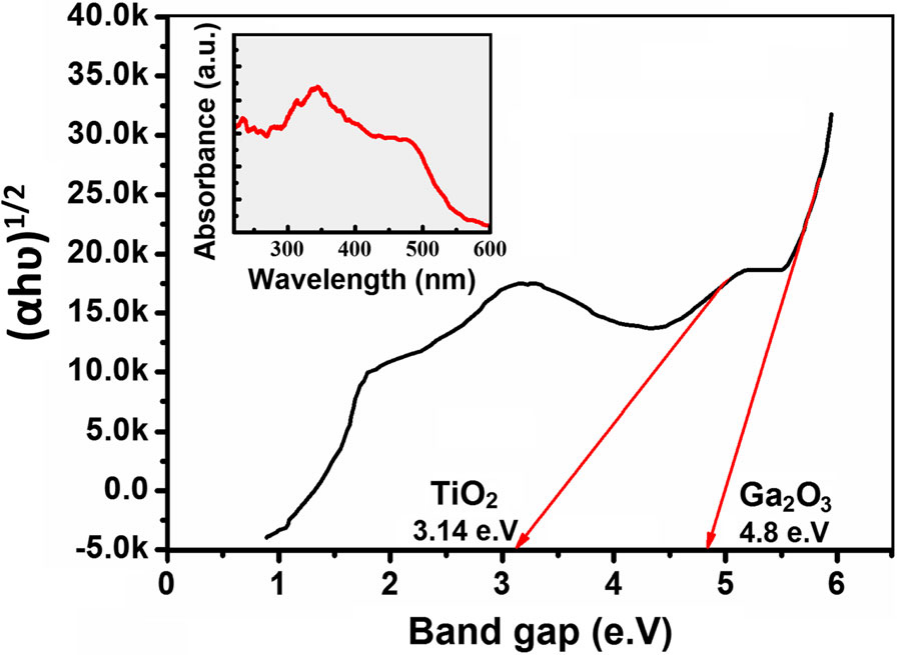
PL spectrum of 2D TiO_2_-Ga_2_O_3_ *n-p* heterostructures at room temperature with bandgaps for TiO_2_ and Ga_2_O_3_

All FTIR spectra of 2D Ga_2_O_3_, TiO_2_ and TiO_2_-Ga_2_O_3_ *n-p* heterostructures are summarized in Fig. 6. As spectra for 2D TiO_2_ and Ga_2_O_3_ are nearly overlapping each other, they therefore were presented separately in Fig 6a and Fig. 6b, respectively, in comparison with the spectrum of 2D TiO_2_-Ga_2_O_3_ heterostructures. The peaks centered at about 1594 cm^−1^ are attributed to the O-H stretching and bending modes of the hydrated oxide surface and the adsorbed water [41]. Moreover, the adsorption of atmospheric CO_2_ on the surface of gallium oxide is characterized by the detection of bands at 1519 cm^−1^ and 1646 cm^−1^, which resulted from preparation and processing of the samples in ambient air [42]. More interesting results were observed in the perturbation area, presented as inserts in Fig. 7a and Fig. 7b, respectively. The IR band at 607.9 cm^−1^ is due to vibration of the Ga-O bond of GaO_6_ octahedra in Ga_2_O_3_ lattice [43]. Its intensity has the maximum in FTIR spectrum of 2D Ga_2_O_3_ and decreased in the FTIR spectrum of 2D TiO_2_-Ga_2_O_3_ *n-p* heterostructure. Compared with the FTIR spectrum of 2D Ga_2_O_3_ nano-film, a new peak at 464 cm^−1^ appeared in FTIR spectrum for ALD-fabricated 2D TiO_2_-Ga_2_O_3_ *n-p* heterostructures. This peak is near overlapping typical characteristic peak at 470 cm^−1^ for TiO_2_ [15].

Photoluminescence (PL) technique is usually employed to investigate the migration, transfer and recombination rate of the photo-induced electrons-holes pairs in semiconductors. Figure 7 shows the room temperature (25 °C) PL spectra of ALD-fabricated 2D TiO_2_-Ga_2_O_3_ *n-p* heterostructures annealed at 250 °C with the details of the measured bandgap for TiO_2_ and Ga_2_O_3_, respectively. There are two peaks in the PL spectra for the 2D TiO_2_-Ga_2_O_3_ *n-p* heterostructures (presented in insert in Fig. 7): one is called near band edge emission (NBE), which is in the UV region due to the recombination of free excitons through an exciton–exciton collision process; and the second one is called deep level emission (DPE), which is caused by the impurities and/or structural defects in the crystal [41]. The DPE intensity in 2D TiO_2_-Ga_2_O_3_ *n-p* heterostructures is lower than that in Ga_2_O_3_ [44], which indicates more efficient transfer and separation of the charge carriers owing to the electron-hole transfer in the heterojunctions between TiO_2_ and Ga_2_O_3_. Noteworthy, the DPE of 2D TiO_2_-Ga_2_O_3_ *n-p* heterostructures is shifted towards the UV region whereas DPE of Ga_2_O_3_ is within the visible light region [44]. In addition, the selected annealing temperature of 250 °C did not allow full crystallization of Ga_2_O_3_ nano-film in the heterostructure, which was reflected by the unchanged value of its bandgap (4.8 eV). However, in our previous investigation, it was found that further increase of the annealing temperature (above 250 °C) of such extremely-thin films causes their disintegration with the following agglomeration of their nano-grains into island-like nanostructure [6]. On the contrary, the bandgap for TiO_2_ slightly changed to ~ 3.14 eV compared to its microstructural counterpart.

Consequently, all the above material characterization experiments clearly confirmed the successful, development of conformal and uniform sub-10 nm TiO_2_-Ga_2_O_3_ *n-p* heterostructures. Thus, these 2D TiO_2_-Ga_2_O_3_ *n-p* heterostructures were ALD-fabricated impurity-free on the wafer-scale and subsequently annealed at 250 ^o^C for the establishment of developed *n-p* nano-interface.

**Fig. 8:**
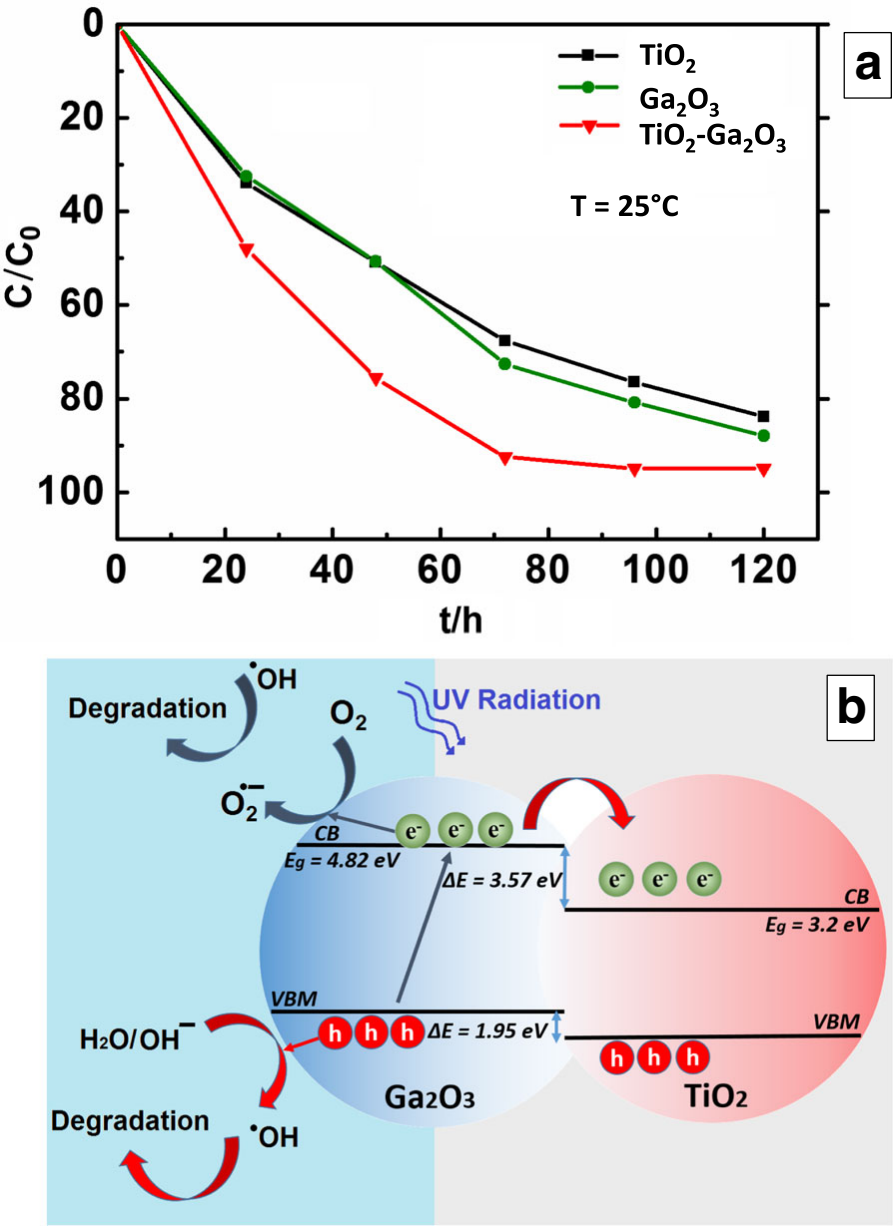
MO degradation efficiency for 2D TiO_2_, Ga_2_O_3_, and TiO_2_-Ga_2_O_3_ *n-p* heterostructures under *λ* = 254 nm UV light (**a**). Schematic photocatalytic reaction process and charge separation transfer of 2D TiO_2_-Ga_2_O_3_ under UV light irradiation (**b**)

The photocatalytic degradation of MO under the UV light irradiation (*λ* = 254nm) was carried out at the room temperature (25 °C) to evaluate the photocatalytic activity of ALD-fabricated 2D TiO_2_, Ga_2_O_3_ and 2D TiO_2_-Ga_2_O_3_ *n-p* heterostructures. As presented in Fig. 8a, 2D TiO_2_-Ga_2_O_3_ *n-p* heterostructure demonstrated higher photocatalytic activity compared to both 2D TiO_2_ and Ga_2_O_3_ under the same UV irradiation. Specifically, using 2D TiO_2_-Ga_2_O_3_ *n-p* heterostructure as the catalyst, MO degradation efficiency reached ~90% within 70 h, while the values for 2D Ga_2_O_3_ and TiO_2_ were approximately ~ 70% and ~ 65%, respectively, at the same time. Considering the fact that 2D Ga_2_O_3_ has not been fully crystallized under the annealing temperature of 250 °C, it is assumed that the weak chemical bond developed between 2D TiO_2_ and Ga_2_O_3_is good enough to ensure the successful role of *n-p* heterojunction for the photocatalytic activity.

The photocatalytic degradation mechanism by 2D TiO_2_-Ga_2_O_3_ *n-p* heterostructure under *λ* = 245 nm UV light irradiation is proposed in Fig. 8b. It is a common knowledge that the photocatalytic degradation of dyes mainly involves several active radical species such as hydroxyl radicals (·OH), holes (h^+^) and electrons (e^−^) [45]. The direct contact between 2D Ga_2_O_3_ and TiO_2_ induced the development of heterojunction owing to the different energy levels. Under *λ* = 254 nm UV light irradiation, both Ga_2_O_3_ and TiO_2_ were excited to generate electrons and holes simultaneously. Large numbers of defects consisting of robust acceptor state in the bandgap trap holes and prevent recombination. Various defect bands promote the electron-hole pair separation rate. The enhanced photo-catalytic performance is mainly derived from the large numbers of acceptor states accompany with Ga_2_O_3_ defects especially in its not fully crystallized phase. The acceptor states not only expand the light absorption edge of UV but also retard the rate of electron-hole pair recombination. In this regard, both large number of defects and acceptor states is responsible for enhancing the photocatalytic performance of 2D TiO_2_-Ga_2_O_3_ *n-p* heterostructure. At the same time, holes in the VB of TiO_2_ can migrate into the VB of Ga_2_O_3_. Thus, the concentration of photo-generated holes on the Ga_2_O_3_ surface increases. The photo-generated holes play a vital role in the photo-degradation process of 2D TiO_2_-Ga_2_O_3_ *n-p* heterostructures. Therefore, the increasing concentration of the photo-generated holes in the VB of Ga_2_O_3_ could also lead to its high photocatalytic activity. Moreover, the higher-specific surface area fabricated after annealing may additionally improve the overall photocatalytic activity of 2D TiO_2_-Ga_2_O_3_ *n-p* heterostructures. The absorption and desorption of molecules on the surface of the catalyst is the first step in the degradation process [46, 47]. Thus, higher surface-to-volume ratio in the surface morphology of the TiO_2_-Ga_2_O_3_ *n-p* heterostructures provides more unsaturated surface coordination sites. Therefore, the annealed 2D TiO_2_-Ga_2_O_3_ *n-p* heterostructures possess higher-specific surface area caused by numerous ultrathin nano-grains, as presented in SEM characterization. Consequently, high surface-to-volume ratio combined with the suitable nano-interfaces obtained for the 2D TiO_2_-Ga_2_O_3_ *n-p* heterostructures resulted in its great photocatalytic activity towards the efficient MO degradation.

## Conclusions

In this work, wafer-scale 2D TiO_2_-Ga_2_O_3_ *n-p* heterostructures with the average thickness of ~ 8.0 nm were successfully fabricated for the first time via a two-step ALD process by using Ti(N(CH_3_)_2_)_4_ and C_33_H_57_GaO_6_ as TiO_2_ and Ga_2_O_3_ precursors, respectively. Their optimal deposition parameters were established. The 2D TiO_2_-Ga_2_O_3_ *n-p* heterostructures were annealed at 250 °C for the structural stabilization and development of the *n-p* nano-interface. Subsequently, 2D TiO_2_-Ga_2_O_3_ *n-p* heterostructures were utilized for efficient MO degradation at the room temperature under the UV light (*λ* = 254 nm) irradiation. 2D TiO_2_-Ga_2_O_3_ *n-p* heterostructures have clearly demonstrated unique capabilities and higher photocatalytic activity than that of pure 2D TiO_2_ and Ga_2_O_3_ for MO degradation. Specifically, the effect of *n-p* heterojunction between *n*-type TiO_2_ and *p*-type Ga_2_O_3_ enabled a higher concentration of the photo-generated holes and larger-specific surface area, which ultimately led to its higher photocatalytic activity. Therefore, sub-10 nm, 2D *n-p* heterostructures can be potentially exploited as promising nano-materials for the practical photocatalytic devices.

## Methods

### Synthesis 2D *n-p* Heterostructure

All reagents and precursors were purchased from the commercial sources and represented analytical grade. They were used as received without further purification. The 4-in. Si/SiO_2_ wafers (12 Ω/cm) were utilized as substrates for ALD depositions, where the thickness of the native oxide was ~ 1.78–1.9 nm. 2D TiO_2_-Ga_2_O_3_ *n-p* heterostructures were prepared by a two-step fabrication method. Prior to ALD depositions, in order to reduce the influence of Si wafer on electrical measurements, an additional ~ 100-nm-thick SiO_2_ insulating layer was applied by CVD, (Oxford Instruments Plasmalab 100). After that 150-nm-thick Au/Cr films were deposited on SiO_2_/Si by the Electron Beam Evaporator method (Nanochrome II (Intivac, USA)) to develop electrodes for subsequent investigations. All ALD fabrications were carried out on Savannah S100 (Ultratech/Cambridge Nanotech). A pulse time of 5 s was used for both the Ga(TMHD)_3_ and O_2_ plasma, at a pressure of 3 × 10^−3^ mbar.

### Characterization

The surface morphology and elemental analysis of ALD-fabricated sub-10 nm TiO_2_-Ga_2_O_3_ heterostructures were characterized by scanning electron microscopy (SEM, SU-500) and energy dispersive X-ray (EDX) spectroscopy (EDS, JEOL). Fourier transform infrared (FTIR) spectra were taken using a NEXUS Thermo Nicolet IR-spectrometer in the range 4000–400 cm^−1^ with a spectral resolution 2 cm^−1^. In order to investigate the surface chemistries of the developed samples, X-ray photoelectron spectroscopy (XPS) was employed in the ESCALAB system with AlK X-ray radiation at 15 kV. All XPS spectra were accurately calibrated by the C1s peak at 284.6 eV for the compensation of the charge effect. Hall effect measurement system (HMS3000) was employed at the room temperature to measure the Hall coefficient of Ga_2_O_3_ thin films by using a 0.55T magnet. EIS and all electrical measurements for 2D TiO_2_, Ga_2_O_3_, and TiO_2_-Ga_2_O_3_ heterostructures were carried out on AutoLab PGSTAT204 (Metrohm Autolab, B.V., Netherlands). Room temperature photoluminescence (PL) spectra of ALD 2D TiO_2_-Ga_2_O_3_ heterostructures were performed on an F-4600 fluorescent spectrophotometer (Hitachi Corp., Tokyo, Japan), and the maximal excitation wavelength was *λ* = 200 nm, and the filter was *λ* = 300 nm. The photocatalytic activity of 2D TiO_2_, Ga_2_O_3_ and 2D TiO_2_-Ga_2_O_3_ heterostructures for the MO (C_14_H_14_N_3_NaO_3_S) degradation in aqueous solution under the UV light was evaluated by measuring the absorbance of the irradiated solution. For this study, 2D TiO_2_-Ga_2_O_3_ heterostructures were placed into 100 mL of MO solutions with a concentration of 6 mg/L and a pH of 6.5. The solutions were continuously stirred in the dark for 2 h before illumination in order to reach the absorption-desorption equilibrium between MO and the 2D TiO_2_-Ga_2_O_3_ heterostructures. Then the solutions were irradiated by a 30 W low-pressure UV lamp (*λ* = 254 nm), which was located at the distance of 50 cm above the top of the dye solution. During the process, 5 mL solutions were pipetted every 12 h for the absorbance determination by a UNIC UV-2800A spectrophotometer using the maximum absorbance at 465 nm. All experiments were performed under the ambient condition and room temperature. The degradation efficiency of MO was defined as

1 $$ D=\left[\left({\mathrm{A}}_0-{A}_t\right)/{\mathrm{A}}_0\right]\times 100\%, $$

where *D* is degradation efficiency, *A*_0_ is the initial absorbance of MO solution, and *A*_*t*_ is the absorbance of MO solution after UV irradiation within the elapsed time *t*.

## Abbreviations

EDS: Energy dispersive spectroscopy
FTIR: Fourier transform infrared
MO: Methyl orange
PL: Photoluminescence
SEM: Scanning electron microscopy
UV-vis: Ultraviolet-visible
XPS: X-ray photoelectron spectroscopy
