# Correction to: Wafer-Scale Fabrication of Sub-10 nm TiO2-Ga2O3 n-p Heterojunctions with Efficient Photocatalytic Activity by Atomic Layer Deposition

**DOI:** 10.1186/s11671-019-3028-5

**Published:** 2019-05-27

**Authors:** Hongyan Xu, Feng Han, Chengkai Xia, Siyan Wang, Ranjith K. Ramachandran, Christophe Detavernier, Minsong Wei, Liwei Lin, Serge Zhuiykov

**Affiliations:** 1 School of Materials Science and Engineering, North University of China, Taiyuan, 030051 People’s Republic of China; 2 Department of Solid State Science, Ghent University, Krijgslaan 281/S1, B-9000 Ghent, Belgium; 3 Berkeley Sensor and Actuator Center, Department of Mechanical Engineering, University of California, Berkeley, CA 94720 USA; 4 Ghent University Global Campus, 119 Songdomunhwa-ro, Yeonsu-gu, Incheon, 21985 South Korea

**Correction to: Nanoscale Res Lett**

**https://doi.org/10.1186/s11671-019-2991-1**

Please be advised that the name of one of the coauthors in the original article [1] has been incorrectly spelled: *‘Ranish M. Ramachandran’* should be *‘Ranjith K. Ramachandran’*.

The authors apologize for this error.
